# Some Remarks on the Destruction of Shade Trees as a Sanitary Measure

**Published:** 1882-01

**Authors:** Rufus W. Griswold

**Affiliations:** Rocky Hill, Conn.


ARTICLE II.

### SOME REMARKS ON THE DESTRUCTION OF SHADE TREES AS A SANITARY MEASURE.

By RUFUS W. GRISWOLD, M. D., of Rocky Hill, Conn.

The matter of the preservation of health by the private or public enforcement of what are called sanitary measures, has of late years begun to assume considerable proportions, and to be the occasion for quite an amount of reading in the press. Not alone in publications particularly devoted to the interests of sanitary engineering and engineers, but in medical journals, and also in the general press—religious, secular, literary, etc., etc.,—a measure of type is devoted to the beneficent purpose of instructing us as to how we shall so take care of our environments, as to escape choleras, dysenteries, diphtherias, typhoids, intermittents, and so on,—the premise assumed being that the large part of these proceed out of vicious surroundings, and are to be prevented by a correction of the faults: further, indeed—that, excepting the fruits of contagion, they all do; and so are controllable, and ought to be utterly stamped out. Some part of the instruction thus magnanimously vouchsafed to us comes from persons who, we may legitimately suppose, ought to know something of the subject they are talking about; but the maximum of it is from writers, tolerably perhaps, and perhaps thoroughly, posted in their own other proper business or calling, but essentially ignorant upon the special matter with which they attempt to wrestle. And when we have put together the untenable deductions of some who ought to know, and the ignorant assertions of vastly more who really know nothing of the matter treated of, it is not surprising that with a few grains of defensible sense we get tons of bosh. To know who it is that understands the subject under treatment and who it is that does not, is difficult; since the clever copyist, adopting the theories and arguments of some one gone before, makes show on paper quite as brilliant as the original, and helps to spread and deepen the force, false or true, of his predecessor's argument. It follows that thus error, quite as often as truth, is not only put forth, but is indefinitely multiplied.

This as preliminary, and in enforcement of our lesson.

In a secular paper of recent date, I observed a second-hand article, based upon a treatise by a sanitary engineer, in which was an onslaught against shade trees, as potent factors of disease. It was calculated to produce more or less of almost irreparable mischief. It opened out with stating that the first instrument of the sanitary engineer should be an axe: it followed with the statement that shade trees about dwellings, encouraging dampness, and keeping out the sun, were the causes of great amounts of sickness of various sorts, and that if we would have immunity from these sicknesses, and become healthy and robust, we should slaughter the trees and let in that blessed renovator and purifier and invigorator of physical life, the light of the God-given sun: no one need expect to be free from typhoids and malarias over whose roof the maple and elm cast their baleful shadows. And so on to quite a lengthy argument. It is worth our while to look a little into the validity of this argument, to enquire upon what it is based ; to eliminate some of the errors out of it and to expose its fallacious deductions. If we can do something of this, it may possibly prevent an occasional individual from allowing himself to be made a fool of by the advice of some other fool who is the possessor of a delusion.

It is not at all necessary undertaking to show that sunlight is not an indispensable article to the earth and those who inhabit it; but it is possible to have too much of it for one's good, and it is not essential to have that too much. When a settler puts up his cabin in the depths of the primeval forests, it is a good thing to clear away the woods from around it ; but it was not of this sort of habitation that the article alluded to, but of the ordinary general condition of dwellings in New England at the present time. Now, we find about many of these more or less of trees, usually transplants, serving for shade and for what we have been wont to consider an agreeable protection against too much sun. It is a solemn fact—we will not undertake to deny it —that the denizens of a great many of these tree-shaded houses have rheumatisms, diarrhœas, diphtherias, malarial troubles, and so on. For illustration, the writer will take his own house. It is in a generally very healthy country village, and favorably situated for drainage. A very careful wife keeps it respectably clean inside, and a passably careful man keeps things ordinarily clean around it. But it is pretty well banked in with trees; in front, towering twins of Norway spruce screen the windows and cover the roof to the ridge board, and three lines of maples, ash and horse-chestnut in close contact considerably more than fill up the gaps. A luxuriant grape vine crawls all over one end to the last clapboard and up to the shingles. Around the other corner are more grape vines, and a splendid fir; and a little further four more specimens of the spruce—compact, thick-grown, splendid types, beneath the heavy shade of which the tenant, in the lazy summer days, swings his hammock, and bethinks himself that but little else is needed, save the essential factor of contentment to bring the hour very near to terrestial paradise. Around the other corner another boastful grape vine covers over the windows of the "Ell," canopies the door, and carpets part of the roof. The house is old, and as it would go for money, cheap ; but it is cosy, comfortable, and well shaded. How are the folks? The man has had typical intermittent, and dumb ague, and bowel complaint in the summer season ; his wife has had malarial troubles and rheumatism and erysipelas ; his sons have had ague, and so also has the chore boy. And in his mind's eye the writer can see a dozen other houses in the village, somewhat similarly environed, where also they have sicknesses, particularly intermittent fever the last few years. Out of the other corner of the same eye are seen, here and there about the town, many houses around which there are no trees, and no other interference with the sun, in which they, the residents, *do not* have ague and fever! From the consideration of these facts, what is to be the conclusion? Clearly this: If here are a dozen houses shaded, and in them there is intermittent, and another dozen houses in the same village not shaded, and in them not any intermittent, then it logically follows that the shade—in other words, the exclusion of the sun light—is at the least a powerful factor in the etiology of the prevailing troubles. The sanitarian, with his axe, has his case made out, on my own showing, and is justified, in behalf of the health of my family, to walk in and fell what I years ago planted, have nurtured, and taken into the essence of my being as a comfort and a joy. But mind you—he won't do it. And I will give abundant reason why I am not to be fooled by his deductions from certain conditions not denied.

*(TO BE CONTINUED.)*

